# Promoter hypermethylation-induced transcriptional down-regulation of the gene *MYCT1* in laryngeal squamous cell carcinoma

**DOI:** 10.1186/1471-2407-12-219

**Published:** 2012-06-06

**Authors:** Min Yang, Wei Li, Yi-Ying Liu, Shuang Fu, Guang-Bin Qiu, Kai-Lai Sun, Wei-Neng Fu

**Affiliations:** 1 Department of Medical Genetics, China Medical University, Shenyang, 110001, P.R. China; 2 Shenyang Police-dog Technology School of Ministry of Public Security, Shenyang, 110034, P.R. China; 3 ENT Department, the First Affiliated Hospital of China Medical University, Shenyang, 110001, P.R. China; 4 Department of Laboratory Medicine, No. 202 Hospital of PLA, Shenyang, 110003, P.R. China

**Keywords:** Hypermethylation, MYCT1, Laryngeal squamous cell carcinoma

## Abstract

**Background:**

*MYCT1,* previously named *MTLC,* is a novel candidate tumor suppressor gene. *MYCT1* was cloned from laryngeal squamous cell cancer (LSCC) and has been found to be down-regulated in LSCC; however, the regulatory details have not been fully elucidated.

**Methods:**

Here, we sought to investigate the methylation status of the CpG islands of *MYCT1* and mRNA levels by bisulfite-specific PCR (BSP) based on sequencing restriction enzyme digestion, reverse transcription and real-time quantitative polymerase chain reaction (RQ-PCR). The function of specific sites in the proximal promoter of *MYCT1* in LSCC was measured by transient transfection, luciferase assays, electrophoretic mobility shift assay (EMSA) and chromatin immunoprecipitation assay (ChIP).

**Results:**

The results suggested hypermethylation of 12 CpG sites of the promoter in both laryngeal cancer tissues and the laryngeal cancer line Hep-2 cell. The hypermethylation of the site CGCG (−695 to −692), which has been identified as the c-Myc binding site, was identified in laryngeal cancer tissues (59/73) compared to paired mucosa (13/73); in addition, statistical analysis revealed that the methylation status of this site significantly correlated with cancer cell differentiation(p < 0.01). The mRNA level of *MYCT1* increased in Hep-2 cells treated with 5-aza-C (p < 0.01). The luciferase activity from mutant transfectants pGL3*-MYCT1m* (−852/+12, mut-695-C > A, mut-693-C > G) was significantly reduced compared with the wild type pGL3-*MYCT1* (−852/+12), while the luciferase activity from wild transfectants pGL3*-MYCT1* (−852/+12) rose after 5-aza treatment in Hep-2 cells. Finally, EMSA and ChIP confirmed that the methylation of the CGCG (−695 to −692) site prevented c-Myc from binding of the site and demethylation treatment of the 5′ flanking region of *MYCT1* by 5-aza induced the increased occupation of the core promoter by c-Myc (p < 0.01).

**Conclusion:**

In summary, this study concluded that hypermethylation contributed to the transcriptional down-regulation of *MYCT1* and could inhibit cancer cell differentiation in LSCC. DNA methylation of the CGCG site (−695 to −692) of *MYCT1* altered the promoter activity by interfering with its binding to c-Myc in LSCC. Epigenetic therapy of reactivating *MYCT1* by 5-aza should be further evaluated in clinical trails of LSCC.

## Background

Laryngeal cancer is the second most common respiratory system cancer, and the overall incidence of laryngeal cancer in individuals between 0–44 years old in China has shown a highly increasing trend in the recent decades [1,2]. Greater than 90% of laryngeal cancer has been pathologically identified as laryngeal squamous cell carcinoma (LSCC) [3]. Currently, the primary treatment for LSCC is surgery followed by radiotherapy [4]. Total laryngectomy, the most common type of surgery used in advanced cases, seriously impairs laryngeal function and the quality of life of LSCC patients [5]. Therefore, understanding the epigenetic pathways involved in the pathogenesis of LSCC is still urgently needed to improve the chemotherapeutic and diagnostic molecular biomarkers to objectively appraise LSCC; in addition, a better understanding of these pathways will improve treatment and increase the survival rate of LSCC patients [6].

*MYCT1*, which was previously named *MTLC* (c-Myc target from laryngeal cancer cells, GenBank access number AF527367), was cloned by our team in 2003. *MYCT1* is located on 6q25 and has two exons. As a putative target of c-Myc, *MYCT1* is expressed in the nuclei of human hepatocellular carcinoma cells, while a wide distribution of *MYCT1* has been demonstrated in various tissues [7]. The closest homolog, mouse MT-MC1 (c-Myc target in myeloid cells-1), can regulate many c-Myc target genes in myeloid cells, which implies that like MT-MC1, *MYCT1* may participate in the regulation of cell function through the c-Myc signaling network [8-10]. We previously found that *MYCT1* is down-regulated in gastric cancer [11]. Recently, Fu *et al.* in our group found that *MYCT1* is transcribed at a low level in LSCC. These authors also confirmed that the transcriptional start site of *MYCT1* is located 140 bp upstream of the ATG start codon using 5' rapid amplification of cDNA ends (RACE)(GenBank access number GU997693.1) and that c-Myc can regulate the promoter activity of *MYCT1* by specifically binding to the E-box elements within the −886 to −655 bp region [12].

c-Myc is a transcription factor that forms heterodimers with the protein Max and binds gene promoters by recognizing the DNA sequence CACGTG, which is called a canonical E-box [13]. c-Myc can also bind several other non-canonical E-box motifs, such as CATGTG, CATGCG, CACGCG, CACGAG, CGCGAG and CAACGTG [14-16]. Understanding the regulation of the expression of c-Myc target genes is an important step for understanding their biological functions in both physiological and pathological contexts [17]. Several studies have demonstrated that methylation of the CpG dinucleotide within the E-box can prevent the access of c-Myc to target gene promoters *in vivo*[18,19]. This idea has been further reinforced by a study that has shown that c-Myc does not bind to EGFR in HL-60 cells due to methylation of the E-box sites, whereas 5-aza-2′-deoxycytidine (5-aza), a demethylating agent that blocks cellular DNA methyltransferase activity, can restore the binding of c-Myc to the E-box and increase gene transcription [20]. Based on these premises, a causal link exists between methylation of the E-box and c-Myc binding and transcription.

We speculate that *MYCT1* might act as a tumor suppressor gene (TSG) in LSCC carcinogenesis and metastasis. The mechanism of abnormal expression of TSGs in the process of malignant transformation primarily involves point mutations, loss of heterozygosity, and epigenetic modifications (such as hypermethylation) [21]. Specifically, aberrant methylation of CpG islands in gene-specific promoter regions can affect transcriptional function and has been found to be related to human diseases [22]. In this study, we sought to investigate the methylation status of CpG islands, mRNA levels and the function of specific promoter sites of *MYCT1* in LSCC.

## Materials and methods

### Cell culture

The human laryngeal carcinoma cell line Hep-2 was obtained from the Cell Biology Institute of Shanghai, Chinese Academy of Science. The cells were grown in RPMI-1640 with 10% FBS, penicillin (100 U/ml) and streptomycin (100 μg/ml) at 37 °C in a 5% CO_2_ humidified atmosphere.

### Samples and tissue DNA extraction

Seventy-three patients who were diagnosed with resectable LSCC tumors at the ENT Department of the 463 Hospital of PLA between 2002 and 2005 were enrolled in the study. The LSCC tissues were pathologically confirmed according to the UICC classification (TNM 2002). Patients who had undergone radiotherapy or chemotherapy prior to surgery were excluded from the study. All of the patients provided written informed consent, and approval for the study was received from the Ethics Committee of China Medical University. All specimens, including cancerous and paired adjacent normal laryngeal tissues, were obtained fresh during the surgery and stored at −80 °C. Genomic DNA from the LSCC tissues and Hep-2 cells was isolated with DNAzol reagent (Invitrogen Life Technologies, San Diego, CA). Purified DNA was concentrated by ethanol precipitation. The DNA concentration was determined using a spectrophotometer.

### Bisulfite modification and bisulfite-specific PCR (BSP)

Genomic DNA from Hep-2 cells and 10 LSCC samples was randomly selected for methylation status screening. Approximately 1 μg of genomic DNA was bisulfite-modified using the EZ DNA Methylation-Gold Kit^TM^ (Zymo Research, Orange, CA) according to the manufacturer's recommendations. Based on the functional promoter sequence of the *MYCT1* gene, the primers (F: 5'-TTAAATAGAGAAATAGATATGTTAAGAATA-3'; R: 5'-TATACAAAATTAAAAAATAAACCAC-3') used in bisulfite-specific PCR (BSP) detection were designed using MethPrimer (http://www.urogene.org/methprimer/index.html), and the amplified fragment was 449 bp. The PCR reaction was performed in a 25 μl reaction system, starting with denaturation at 94 °C for 4 min, then 30 cycles of denaturation at 94 °C for 30 sec, annealing at 53 °C for 30 sec, extension at 72 °C for 45 sec, followed by an extra extension at 72 °C for 5 min. The BSP products (200 ng) were then cloned into a T-vector (TaKaRa, Japan), and JM109 *E. coli* competent cells (TaKaRa, Japan) were used for transformation according to the manufacturer’s instruction. The sequence of the BSP product was obtained using the ABI model 3730 sequencer. According to the previous study, full methylation at a particular CpG island was defined as follows: >60% of the average bisulfite sequencing signal was “C,” whereas partial methylation was 40% to 60%. “Methylation-positive” was defined as at least 6 of the 12 CpGs within the BSP product showing full or partial methylation [23]. We then detected the methylation status of the CGCG site within the non-canonical E-box motif binding to c-Myc, CACGCG, in other LSCC tissues using BSP-based RFLP by cutting the *AccII* site. Briefly, 8.5 μl of BSP product digestion was initiated by adding 1 μl of 10X Buffer and 0.5 μl of 10 U/μl *AccII*(TaKaRa, Japan) for 6 to 7 hours in a 37 °C water bath, 5 μl of the product was analyzed on 2.5% agarose gels and the gel image was visualized under a GDS8000 (UVP, USA).

### Reverse transcription and real-time quantitative polymerase chain reaction (RQ-PCR)

After culture in a 6-well plate for 24 h, Hep-2 cells were treated with 4 μM 5-aza (Sigma Chemical Co.) for 3 days as previously described [23]. BSP was used again to detect wether Hep-2 cells had been demethylated or not. Total RNA was isolated from Hep-2 cells and Hep-2 cells treated with 5-aza using Trizol reagent (Invitrogen, USA) following the protocol. cDNA was synthesized by reverse transcription using an AMV RNA PCR kit (TaKaRa, Japan). The *MYCT1* primers for RQ-PCR were F: 5'- AGGGAGTCCATGGCCAGAAA -3' and R: 5'- ATGAACACAGCCCAAATAAATCCTC -3', which amplified a 105 bp product. GAPDH served as an internal control, which primer sequences were 5'-TGCACCACCAACTGCTTAG -3' and 5'- GACGCAGGGATGATGTTC -3' and were expected to produce a 175 bp DNA fragment. RQ-PCR was carried out on a real-time quantitative PCR instrument (ABI, USA) using a TaKaRa SYBR® Premix Ex Taq™ kit (TaKaRa, Japan). The 20-μl reaction system comprised 400 nM primers, 50 ng cDNA and 10 μl SYBR Premix Ex Taq™. The cycling was designed as 95 °C for 30 s, 95 °C for 5 s, and 60 °C for 34 s for 50 cycles. Each sample was measured in 3 separate tubes, and each experiment was run in triplicate. Results of real-time PCR were processed using a ΔΔCt method [24]. cDNA from untreated Hep-2 cells was used as calibrator sample.

### Plasmid construction and site-directed mutagenesis

We obtained a novel transcript variation of *MYCT1* using 5' RACE, named c-Myc target 1 transcript variant 1(GenBank access number GU997693.1), which indicated the transcript start site of *MYCT1* and its variation. Three 5' deletion constructs of p852 (−852/+12), p799 (−799/+12), and p667 (−667/+12) of the *MYCT1* promoter region were generated by our group, and the results from dual luciferase assays displayed that P852 (−852/+12) acted as the proximal promoter [12]. Based on the wild-type construct of p852, the P852 mutant was created to replace the E-box B core sequence "CACGCG" with "CAAGGG" (named P852-mutB) using the Gene Tailor site-directed mutagenesis system (Invitrogen, USA) according to the manufacturer's instructions. The primers Fp (forward primer containing site *Sac*I, 5'-TTTGAGCTCGTGGCCGAGCGCAGT-3'), Rp (reverse primer containing site *Hind*III, 5'-TTTAAGCTTGTTATTAGCCATAATATCCACAAGA-3'), fm (mutated forward primer, 5'-GTGTGGTGGCAAGGGCCTGTAATCCC-3') and rm (mutated reverse primer, 5'-GGGATTACAGGCCCTTGCCACCACAC-3') were used in site-directed mutagenesis. The pMD^TM^-T Vector (TaKaRa, Japan) was used for TA cloning and JM109 *E. coli* competent cells (TaKaRa, Japan) were used for transformation. Colonies were selected and rechecked by electrophoresis. The authenticity of all constructs was confirmed by DNA sequencing on an ABI 3730xl 96-capillary DNA analyzer (ABI, US).

### Transient transfection and luciferase assays

All of the cells used for the subsequent experiments were in log phase. Hep-2 cells were cultured in 24-well plates at a density of 1 × 10^5^ per well overnight in RPMI-1640 without FBS and antibiotics. P852, P852-mutB or the empty construct of the luciferase reporter gene (0.8 μg) and 16 ng of the internal control plasmid pRL-TK were co-transfected into Hep-2 using Lipofectamine™ 2000 (Invitrogen, USA). The relative luciferase activity was normalized to the TK activity value. At 6 h after transfection, RPMI-1640 with FBS and antibiotics was added to the cells. The cells were harvested at 48 h after transfection, and the lysates were analyzed for luciferase activity using the Dual Luciferase Reporter assay (Promega) in a GloMax™ Microplate Luminometer (Promega). Hep-2 cells pre-treated with 5-aza (4 μM, 3 days) were used for P852 to be transfected. The results were expressed as relative luciferase activity. The data from triplicates out of five replicates from which the largest and the smallest values were excluded from the analysis are represented as the mean ± SD.

### EMSA (electrophoretic mobility shift assay)

Two sets of complementary oligonucleotides, F: 5’-CGGGTGTGGTGGCACGCGCCTGTAATCCCA -3’ and R: 5’-TGGGATTACAGGCGCGTGCCACCACACCCG-3’ including the core sequence of CACGCG(−697 to −692), were labeled by Biotin 3' End Labeling Kit (Pierce, USA). EMSA used the Lightshift Chemiluminescent EMSA kit (Pierce, USA) according to the protocols. Nuclear extracts were prepared from Hep-2 cells using a nuclear extract kit (Active Motif, USA) as described in the instructions. The methylated probe was obtained by incubating 20 μg of the oligonucleotide with 80 units of M.SssI CpG methylase for two hours at 37 °C *in vitro*. Then, the mixture was heated at 65 °C for 20 min to inactivate the methylase, purified by polyacrylamide gel electrophoresis, and concentrated with Centricon 3 microconcentrators. The two types of probes (1 ng) were incubated with nuclear extracts (6 μg) in protein-DNA binding buffer (2 μl of 10X binding buffer, 1 μl of 50% Glycerol, 1 μl of MgCl_2_, 1 μl of poly(dI–dC), and 1 μl of 1% NP-40) on ice for 20 min. Competition reactions were pre-performed with a 40/100-fold molar excess of unlabeled, methylated double-stranded competitor DNA. For the supershift reaction, 1 μg of each anti-c-Myc antibody (N-262X, Santa Cruz) was preincubated with the nuclear extracts in the absence of poly (dI.dC) for 20 min at 4 °C. Subsequently, poly (dI.dC) was added and incubated for 5 min, followed by the addition of labeled probe for an additional 20 min. The DNA-protein complexes were separated by electrophoresis on a 6% native polyacrylamide gel for 1.5 to 2.5 h at 4 °C and 100 V, and the complexes were visualized by autoradiography.

### Chromatin immunoprecipitation assay (ChIP)

ChIP assay were performed as previously described [12]. Hep-2 cells were treated with 5-aza in accordance with RQ-PCR. Cells were fixed by the addition of 1% formaldehyde in growth medium for 10 min at 37 °C. After washed with ice-cold PBS, cells were lysed with lysis buffer containing protease inhibitors and chromatin from cells was sheared by sonicator. Half of DNA fragments was saved as input DNA. The rest was incubated with anti-c-Myc antibody (N-262X, Santa Cruz), and immunoprecipitated by protein A/G plus-agarose (Santa Cruz, USA) which was eluted and extracted with phenol-chloroform, whose concentrations were measured with a spectrophotometer. The input and immunoprecipitated DNA were used as templates for Real Time PCR performed with *MYCT1* core promoter-specific primer amplifying the second c-Myc binding regions (c-Myc B, F:5'-GAGGTCAGGCCTAGTTCATG-3', and R:5'-CTTAGT CTCGCTCTGTCGC-3') in accordance with RQ-PCR except the difference of 40 cycles. The percentage of *MYCT1* promoter that was bound with c-Myc B was calculated based on the differences between Ct values for input and DNA samples immunoprecipitated from Hep-2 cells treated or untreated with 5-zaz [25].

### Statistical analysis

All statistical analyses were performed using the SPSS 13.0 software. The chi-squared test was utilized to analyze the relationship between methylation and clinicopathological characteristics. The comparisons mRAN level and percentage of MYCT1 promoter occupation were carried out using one-way analysis of variance (ANOVA) and the Students-Newman-Keuls (SNK) method were employed for group comparison. *P* values of less than 0.05 were considered to be statistically significant.

## Results

### Methylation status of the *MYCT1* promoter region

**Figure 1:**
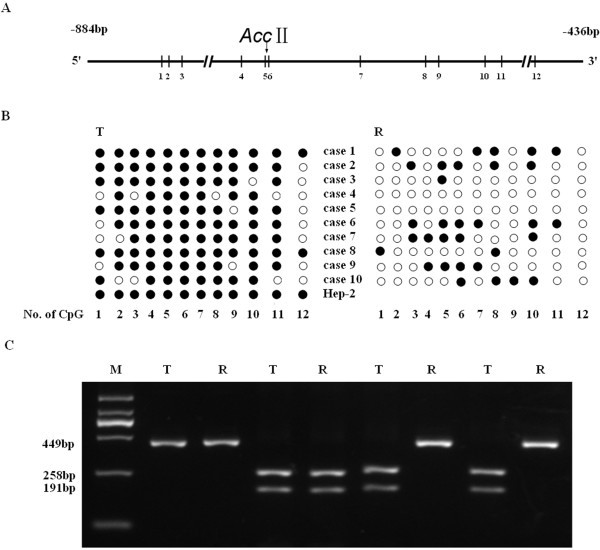
** Detection of promoter methylation of* MYCT1 *in LSCC and Hep-2 cells. A**. Schematic representation of the location of the CpG sites and the AccII restriction site in the *MYCT1* gene. Arabic numerals 1 to 12 represent the number of CpG dinucleotides in the region. **B**. Statistical result of DNA methylation in 10 LSCC paired samples and Hep-2 cells by BSP-based sequencing. **C**. Representative electrophoresis image of paired LSCC samples by BSP-based RFLP.

Twelve CpG sites are illustrated in the sequence of the PCR product (Figure 1A). BSP-based sequencing showed that 11 of the 12 CpG sites in the *MYCT1* gene promoter displayed full methylation except site 12 in Hep-2 cells and LSCC, which indicated that the *MYCT1* promoter was methylation-positive (Figure 1B). Both BSP-based sequencing and RFLP results (Figure 1C) at the site CGCG (−695 to −692) revealed that 46 of 73 (63%) cases of LSCC showed methylation in cancer tissues and no methylation in paired normal tissue, 13 cases showed methylation in both cancer and paired normal tissues, and 14 cases showed no methylation in either cancer or paired normal tissues. These data indicated that a significant difference in *MYCT1* promoter methylation status exists between cancer and paired normal tissues (Table 1). Tumors classified as undifferentiated or poorly differentiated compared with those classified as well differentiated showed significantly higher methylation. The *MYCT1* hypermethylation in LSCC was not significantly associated with age, gender, TNM staging, lymph node metastasis, distant metastasis or clinical stage of the patients (Table1).

**Table 1:** Correlation of CGCG (−695~-692) methylaton with case characteristics

	**Case (n)**	**Positve (n)**	**Negative (n)**	***P***
Sample				
Cancer tissue	73	59	14	P<0.01
Paired normal tissue	73	13	60	
Age at surgery				
<60	35	28	7	0.864
>60	38	31	7	
Gender				
Male	58	47	11	0.928
Female	15	12	3	
Histological classification				
Poorly differentiated	49	46	3	P<0.01
Well differentiated	24	13	11	
T classification				
T1	13	10	3	0.980
T2	26	21	5	
T3	28	23	5	
T4	6	5	1	
Lymph node metastasis				
Negative	55	44	11	0.755
Positive	18	15	3	
Distant metastasis				
Negative	69	56	13	0.761
Positive	4	3	1	
Clinical stage				
I	13	9	4	0.533
II	22	17	5	
III	30	26	4	
IV	8	7	1	

### Effects of treatment with epigenetic-modifying agents on *MYCT1* mRNA expression

**Figure 2:**
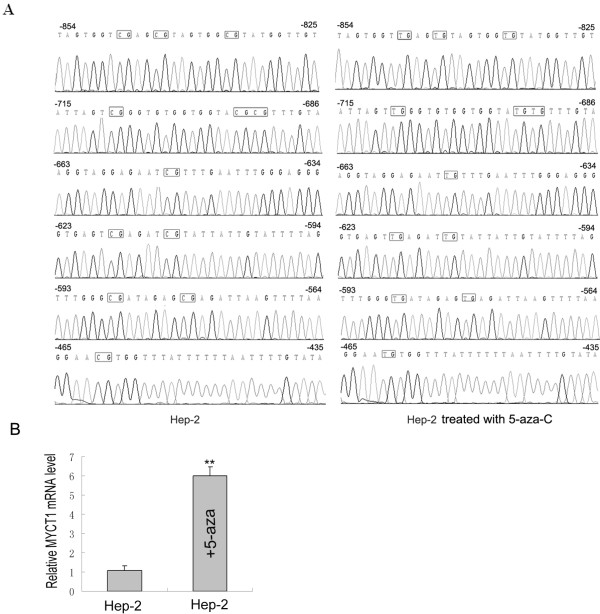
** Effect of 5-zaz on* MYCT1 *expression in Hep-2 cells**. **A.** BSP-based sequencing result of all of 12 CpG sites methlated or demethylated of in Hep-2 cells of the absence or presence of 5-aza. **B**. *MYCT1* mRNA levels in Hep-2 cells. +5-zaz indicates Hep-2 ells were treated with 5-zaz; *MYCT1* mRNA levels were significantly different.** p < 0.01.

To investigate the possible epigenetic regulation of *MYCT1* expression, RQ-PCR analysis was performed in Hep-2 cells that did or did not be treated with a DNA demethylating agent, 5-aza. BSP-based sequencing revealed that the *MYCT1* gene promoter was demethylated in Hep-2 cells by the treatment of 5-aza (Figure 2A). Following 5-aza treatment of Hep-2 cells, *MYCT1* mRNA expression increased, which showed statistically significant differences among the two groups (p < 0.01) (Figure 2B). This result suggested that DNA methylation modification down-regulated *MYCT1* gene expression.

### The site CGCG (−695 to −692) is involved in *MYCT1* promotion/enhancement

**Figure 3:**
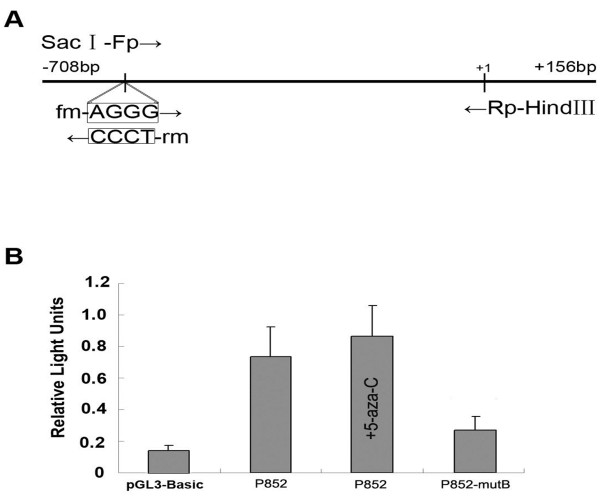
** Effect of mutation and methylation of site CGCG (−695~-692) on* MYCT1 *promoter activity in Hep-2 cells. A**. Schematic representation of the plasmid constructs P852-mutB created by site-directed mutagenesis. The arrows indicate the primers used for constructing the mutant construct. **B**. Luciferase activities in Hep-2 cells of the presence or absence of 5-aza transfected with wild-type and mutant vectors of *MYCT1*.

The mutated sites of P852-mutB are illustrated in Figure 3A. The promoter region between -852 bp and -667 bp was basal for the transcriptional activity, in which the putative core promoter sequences (−730 bp to -681 bp) owned only one DNA motif of regulatory elements, a noncanonical E-box “CACGCG” [7,12]. To determine whether the CGCG site (−695 to −692) affected the promoter activity of *MYCT1*, we created a plasmid containing mutations in the CGCG site (−695 to −692), named P852-mutB, based on the wild-type P852 (−852 to +12). The results from a transient transfection assay showed that the P852 construct expressed a high level of luciferase activity in Hep-2 cells treated with 5-aza, indicating that some important element in the construct up-regulates *MYCT1* expression. The luciferase activities in P852-mutB transfectants were significantly reduced compared with wild-type P852, which suggests that this site plays an important role in regulating *MYCT1* expression (Figure 3B).

### Methylation interferes with the binding between the *MYCT1* promoter and c-myc

**Figure 4:**
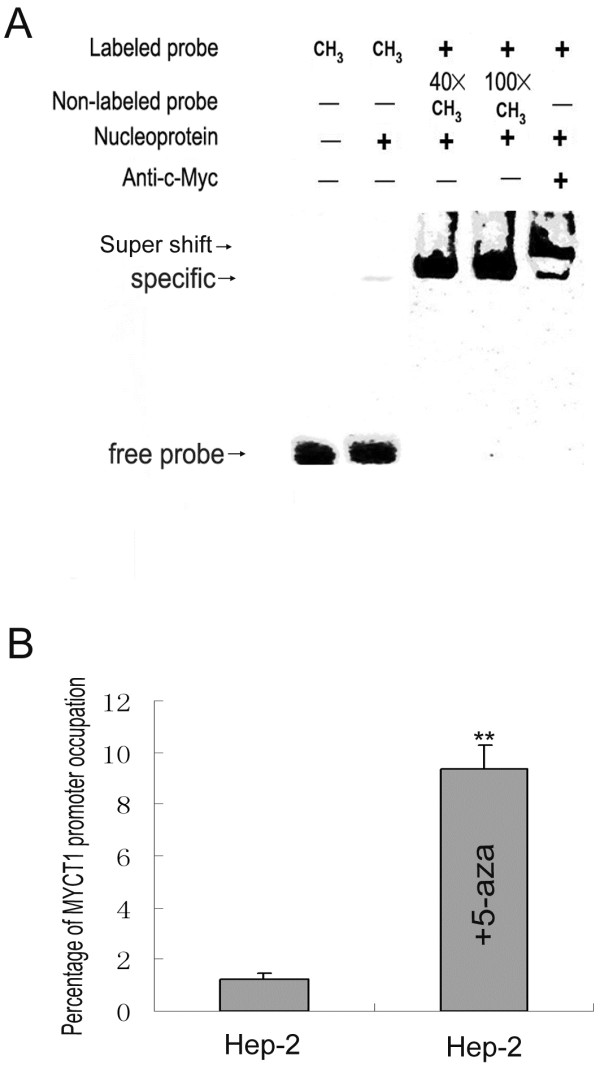
** Effect of methylation of site CGCG (−695~-692) on* MYCT1 *promoter banding to c-Myc in Hep-2 cells. A.** Binding ability of site CGCG (−695~-692) to c-Myc. End-labeled M.SssI-treated methylated and unmethylated *MYCT1* promoter region probes containing CGCG (−695~-692) were mixed with nuclear proteins from Hep2 cells and separated on a 6% polyacrylamide gel. Lane 1, end-labeled M.SssI-treated methylated free probe; Lane 2, end-labeled M.SssI-treated methylated probe mixed with nuclear proteins; Lane 3,4, the unlabeled M.SssI-treated methylated wild-type competitor DNA was mixed with the nuclear proteins before adding end-labeled probe; Lane 5, c-Myc antibody was mixed with the nuclear proteins before adding end-labeled probe; **B**. Binding of E-box B to c-Myc *in vivo* detected by ChIP. The percentage of the *MYCT1* promoter occupied by c-Myc was significantly different in Hep-2 cells of present or absent 5-zaz..** p < 0.01.

EMSA confirmed the specific binding by c-Myc antibody abrogating the band gel shift and the specific shift band was faint when the biotin-labeled probe methylated using M.SssI methylase mixed with nuclear proteins from Hep2 cells (Lane 2, 5 in Figure 4A). In the competition reaction, the specific band was not effectively competed when an excess amount of free probes methylated were added before the binding of nuclear proteins (Lane3,4 in Figure 4A). The effect of promoter methylation on the interaction between c-Myc and *MYCT1* core promoter was further confirmed by ChIP assay. Using the primer to amplify c-Myc B binding regions of *MYCT1* promoter, we observed different DNA levels based on the precipitate by c-Myc antibody from Hep2 cells and Hep2 cells of 5-aza treatment (Figure 4B). The quantities of DNA precipitated by c-Myc antibody from the treated Hep2 cells increased significantly (p < 0.01). These data suggest that the methylation in the core sequence including the CGCG site (−695 to −692) interferes with the specific binding of c-Myc.

## Discussion

Multiple genetic and epigenetic alterations in tumor suppressor genes and oncogenes are important in carcinogenesis [26-28]. Therefore, identifying the corresponding genes and exploring the regulatory mechanisms involved in tumor progression have become critical issues. DNA methylation is one of the main mechanisms of epigenetics. Methylation in gene promoter regions has been well established to contribute to the inactivation of tumor suppressors [29-31].

Our previous study revealed that low *MYCT1* expression is found in LSCC and that *MYCT1* can inhibit cell growth and promote cell apoptosis, which suggests that *MYCT1* may be a candidate TSG [11]. Methylation of CpG islands has been expected in the *MYCT1* promoter region. The present study focused on the epigenetic alterations leading to transcriptional down-regulation of *MYCT1,* thereby opening a new avenue to understand the molecular pathways in laryngeal cancer and to develop potential therapeutics against laryngeal cancer.

In this study, 12 potential methylated sites in the *MYCT1* promoter region were predicted *in silico*, and hypermethylation was observed in 10 LSCC tumor samples and the Hep2 cancer cells by BSP-based sequencing. Among these methylated sites, the sequence CGCG (−695 to −692) is localized in a non-canonical E-box motif, CACGCG, which specifically binds to the transcription factor c-Myc [12]. We asked whether methylation of the potential c-Myc binding site plays a role in the regulation of *MYCT1*. We further explored the methylation status of CGCG (−695 to −692) using BSP-based RFLP in the subsequent 63 tumor samples. In total, 80.8% of the tumor samples showed methylation at this site compared to 17.8% in paired mucosa, which displayed a significant difference. We observed that methylation of the CGCG site (−695 to −692) significantly correlated with cancer cell differentiation but did not correlate with age, gender and TNM staging, suggesting that *MYCT1* methylation could inhibit cancer cell differentiation. In our group, Fu *et al.* found that the average transcription levels of *MYCT1*/*Beta-actin* were 0.42 and 0.83 in 73 cases of laryngeal cancer tissue and paired normal tissue, respectively [12]. We then analyzed the relationship between the methylation status of site CGCG (−695~-692) and the transcription levels of *MYCT1*. An inverse correlation between the methylation status of this site and the *MYCT1* mRNA level was pronounced and significant in either LSCC tissues or paired tissue, which implies that promoter methylation inhibits the transcription of *MYCT1*(Table 2).

**Table 2:** **Difference between CGCG (−695~-692) methylation and mRNA level of* MYCT1 *in LSCC**

	**Methylation status**	**Methylation number (%)**	**mRNA level** ***MYCT1***/**Beta actin (ratio)**	**t value**	**P**
Cancer tissue	+	59	0.31 ± 0.09	18.620	P < 0.01
	-	14	0.88 ± 0.15		
Paired tissue	+	13	0.25 ± 0.11	14.289	P < 0.01
	-	60	0.96 ± 0.17		

To confirm that the aberrant methylation of *MYCT1* induced transcriptional down-regulation, we compared its mRNA levels in laryngeal carcinoma Hep2 cells to Hep2 cells treated with the inhibitor of the methylase enzyme 5-aza. We found the *MYCT1* mRNA levels of *MYCT1* were markedly raised (p < 0.01) after the treatment, suggesting that the gene *MYCT1* was suppressed by epigenetic modification. The mechanism were found in certain tumor suppression genes of cancer cell lines [32,33].

To explore how and why methylation of the CGCG site (−695 to −692) leads to low transcription of *MYCT1*, we constructed the wild-type and mutant-type expressing vectors P852 (−852/+12) and P852-mutB. The results from luciferase assays revealed that the promoter sequence including the non-canonical E-box motif CACGCG (−697 to −692) can have promoter activity and that the point mutation of cytosine at −695 and −693 impaired the activity of luciferase, suggesting that aberrations, including methylation, on this site can affect the promoter activity of *MYCT1*. Interestingly, with the action of 5-aza, P852 owned the higher promoter activity. In further investigation, a CGCG-containing probe was used in EMSA to identify its binding to c-Myc. The result confirmed that the non-canonical E-box motif CACGCG (−697 to −692) could bind to the nuclear extracts, the specific binding band lessened significantly because the probe was methylated, while normal labeled probes could not be competed off by the unlabeled methylated wild-type probe. In addition, we observed DNA demethylation was associated with a rising occupancy percentage of the promoter by c-Myc in ChIP assay. These data suggest that methylation around the CGCG (−695 to −692) in the *MYCT1* promoter might interfere with the binding of c-Myc to this motif. According to a previous report, c-Myc controls the methylation status of the core promoter of target genes through a pathway in which DNA methyltransferase 3A is recruited depending on the cellular context [33]. In addition, the cooperation of c-Myc and other proteins participates in the stable methylation-dependent repression of downstream genes [34]. Consistent with our study, c-Myc could mediate transcriptional silencing through DNA methylation in cell differentiation in cancer [35,36].

5-aza was used to select genes for early detection and prognostic biomarkers of head and neck squamous cell carcinoma (HNSCC) [37]. Recently, 5-aza combined with other therapeutics including irradiation and chemotherapy has been considered and tried in the treatment of LSCC [38,39].

## Conclusions

This report is the first to describe transcriptional down-regulation of the *MYCT1* gene through epigenetic pathways in malignant tumors. We conclude that *MYCT1* is down-regulated by promoter hypermethylation. Our data further indicate that the transcription factor c-Myc participates in the regulation of *MYCT1* transcription and that DNA methylation interferes with the binding of c-Myc to the *MYCT1* promoter region. This result provides a clue for selection of LSCC patients in 5-aza-mediated treatment, but how to solve it is also a problem in further study.

## Competing interests

The authors declare that they have no competing interests.

## Authors’ contributions

MY performed experiments and WL drafted the manuscript. Y-Y L, SF, G-B Q, K-L S and W-N F participated in the design of the study and statistics of data. All authors have read and approved the final manuscript.

## Pre-publication history

The pre-publication history for this paper can be accessed here:

http://www.biomedcentral.com/1471-2407/12/219/prepub
